# Reducing the Standard Deviation in Multiple-Assay Experiments Where the Variation Matters but the Absolute Value Does Not

**DOI:** 10.1371/journal.pone.0078205

**Published:** 2013-10-30

**Authors:** Pablo Echenique-Robba, María Alejandra Nelo-Bazán, José A. Carrodeguas

**Affiliations:** 1 Instituto de Química Física Rocasolano, Consejo Superior de Investigaciones Científicas, Madrid, Spain; 2 Instituto de Biocomputación y Física de Sistemas Complejos, Universidad de Zaragoza, Zaragoza, Spain; 3 Zaragoza Scientific Center for Advanced Modeling, Universidad de Zaragoza, Zaragoza, Spain; 4 Departamento de Fsica Teórica, Universidad de Zaragoza, Zaragoza, Spain; 5 Unidad Asociada IQFR-BIFI, Madrid-Zaragoza, Spain; 6 Fundación Gran Mariscal de Ayacucho (Fundayacucho), La Urbina, Venezuela; 7 Departamento de Bioqumica y Biología Molecular y Celular, Universidad de Zaragoza, Zaragoza, Spain; La Jolla Institute for Allergy and Immunology, United States of America

## Abstract

When the value of a quantity 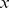 for a number of systems (cells, molecules, people, chunks of metal, DNA vectors, so on) is measured and the aim is to replicate the whole set again for different trials or *assays*, despite the efforts for a near-equal design, scientists might often obtain quite different measurements. As a consequence, some systems’ averages present standard deviations that are too large to render statistically significant results. This work presents a novel correction method of a very low mathematical and numerical complexity that can reduce the standard deviation of such results and increase their statistical significance. Two conditions are to be met: the inter-system variations of 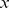 matter while its absolute value does not, and a similar tendency in the values of 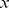 must be present in the different assays (or in other words, the results corresponding to different assays must present a high linear correlation). We demonstrate the improvements this method offers with a cell biology experiment, but it can definitely be applied to any problem that conforms to the described structure and requirements and in any quantitative scientific field that deals with data subject to uncertainty.

## Introduction

Assume a given quantity 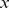 is measured in the laboratory for six different systems (from system 1 up to system 6) where they could be everything from cell types to people or proteins to DNA vectors and even the same system at different points in time (whenever the quantity 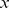 is expected to evolve in some reproducible manner). And as any scientist who wants to be sure he makes no mistakes, the whole set of six measures are repeated three times, say, at different times in different days.

**Figure 1:**
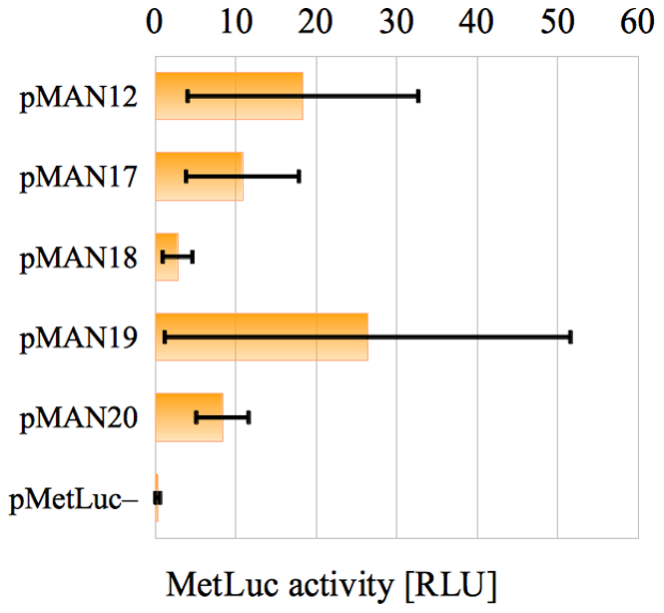
Errors in the starting results. Bar chart representation of the average values 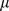 (orange bars) and the associated standard deviation 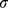 (black capped lines) in tab. 1.

We will call each one of these repeated experiments *assays*, namely, assay 1, assay 2 and assay 3. At the end of the process, we are in possession of 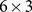 values of the quantity 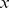; six per each assay, three for each system. Now imagine we obtain the values in table 1 (the odd names given to the six systems in the first column will be later explained). The first thing we can notice about the results is that they do not look right at all. The standard deviation from the average is comparable to the average itself for most of the systems, and only on a couple of them you are “lucky” enough in that the former is about half the value of the latter. When we check the corresponding chart in fig. 1, we run into the same despairing situation. The error bars are huge!

**Table 1:** Starting results.

	assay 1	assay 2	assay 3	*μ*	±	*σ*
pMAN12	33.88	5.65	15.53	18.36	±	14.33
pMAN17	17.60	3.61	11.29	10.83	±	7.01
pMAN18	4.62	0.94	2.72	2.76	±	1.84
pMAN19	55.35	9.30	14.52	26.39	±	25.22
pMAN20	11.15	4.78	9.10	8.35	±	3.52
pMetLuc–	0.00	0.39	0.54	0.31	±	0.28

Activity of the MetLuc protein (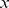 quantity) under the control of six different promoter sequences (the six systems) measured in three assays. The last two columns correspond to the average 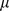 of the three assays for each system, and the associated standard deviation (or error) 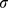. The units as well as the rest of the experiment’s details are described in The experiment.

**Figure 2:**
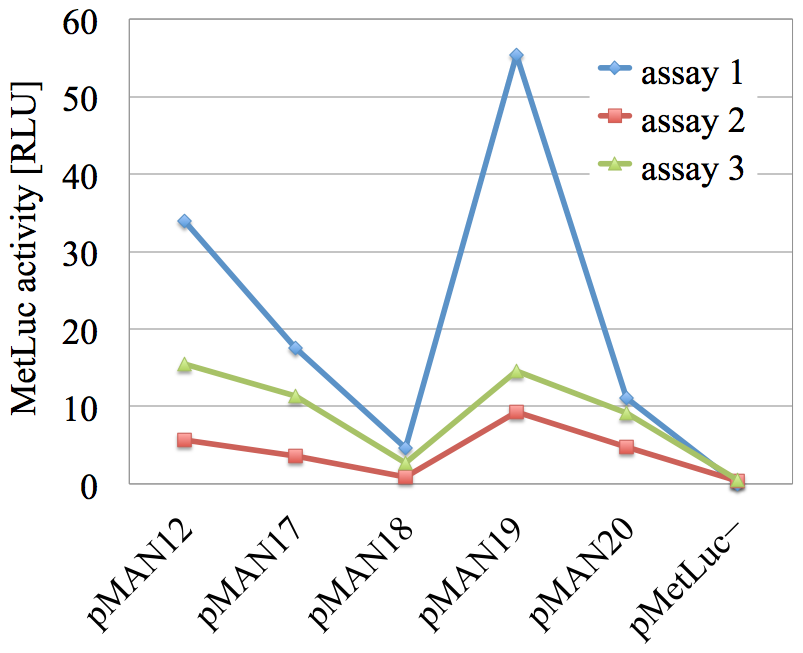
Tendency in the starting results as captured by the different assays. Variation of the quantity 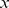 (MetLuc activity) in table 1 for the six systems (vectors) studied. Each color corresponds to a different assay, and the lines joining the experimental points have been added for visual comfort.

Before throwing in the towel, we realize two things about our experiments that might save our day:

- The fact that the *absolute value* of 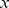 for each given system is not really of much importance but rather the *variation* that 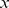 suffers from one system to another, such as whether or not you could safely claim that the value of 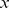 corresponding to system 1 is larger than, and approximately the double of that associated to system 5.
- And that although you seem to be measuring huge differences in absolute value across different assays, it looks as if the “tendency” of the variations is similarly captured in all three of them. This is even more apparent in the graphical representation in fig. 2.

In this work, besides arguing that we were right in not throwing in the towel in such circumstances, we will be interpreting the structure of the results as being caused by a *multiplicative systematic error* (across the different assays). We will also suggest a method to *correct* experimental results in a way where systematic errors may be removed. As a result, the corrected numbers will not tell us anything significant about the “true” absolute value of 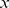 for the different systems. In return, they will maximally capture the tendency that we seemed to be correctly measuring, namely, the averages of the corrected results will present appreciable smaller standard deviations while still follow an average tendency of variation.

In the next section, we will make this precise by introducing the general method of correction as well as a real experiment in cell biology which suffers from the problems (and the virtues!) that we have mentioned in this introduction. In Results, we will apply the correction method to this experiment to show that both the standard deviations and the statistical significance of the results improves considerably. In Discussion, we will discuss our interpretation of the studied situation and the suggested method, compare it to a simpler alternative, and try to explain the surprising fact that something so straightforward cannot be found (as far as we are aware) in the previous literature. Finally, in Conclusions, we will briefly summarize the main conclusions of this work, outline some open questions and suggest lines of future research.

## Methods

### Experimental Setup

As we advanced we will have in general 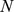 systems, among which a specific one may be called *system* 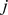, with 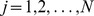. We now measure a quantity 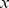 for each one of the 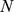 systems, and we repeat 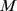 times the whole set of 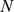 measures. A generic repetition is termed *assay* 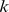, with 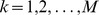, and each one of them is carried out under expected identical conditions. It is convenient to use 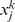 to denote the value of the quantity 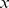 measured for system 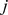 in the 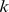-th assay (e.g., in table 1, 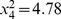).

Each of the different systems can be anything from cities to DNA sequences, from people to chunks of metal and can even be the same system at different points in time if the quantity 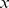 is expected to evolve in some reproducible manner. The differences among the assays may appear due to the experiments being performed by the same researcher at different days, by different but equally skilled researchers using the same equipment or by the same researcher using different (but in principle equally accurate) equipment. It can even be due to different (but in principle equally proficient) laboratories but, as long as we expect different assays to yield same results, their definition is compatible with what we perform here.

For example, the different assays in table II of [1], where the production of four isoforms of *Monilophthora perniciosa* chitinase is presented, do not qualify as the setup described here. The reason for it is simple, as they are *knowingly* carried out at different pH and temperature and therefore they are naturally expected to yield different results.

The experimental setup is thus very general, but we will introduce the correction method as we apply it to a specific example of a *real experiment* in cell biology.

### The Experiment

The aim of the experiment is to elucidate the regulatory network of the human protein called *mitochondrial carrier homolog 1* (Mtch1), and also *presenilin 1-associated protein* (PSAP). Although this protein has been known for almost 15 years to be involved in apoptosis [2] and a number of studies have probed its cellular function [3]–[7], not all the details are known, especially those concerning its regulation, which is uncharted territory at present.

To identify binding sites for transcriptional regulators at the Mtch1 promoter region, different DNA vectors have been constructed and transfected into *Human embryonic kidney 293T* (HEK-293T) cells. Each one of the vectors contains a part of the Mtch1 promoter attached to a *Metridia luciferase* (*MetLuc*) reporter gene. When each vector is transfected into the HEK-293T cells, the MetLuc protein is produced and secreted to the medium, where its activity has been measured using the Ready-to-Glow Dual Secreted Reporter Assay Kit (Clontech). Part of this protocol involves co-transfecting each time with a vector containing the *secreted alkaline phosphatase* (*SEAP*) gene under the control of an early SV40 virus promoter. The SEAP protein is also secreted to the medium, and the measure of its activity is used to normalize the activity of MetLuc. This is done with the objective of eliminating differences in the signal due to changes in the transfection efficiency. Hence, the activity of MetLuc is divided by that of co-transfected SEAP, and the results are reported in *relative light units* (RLU), which are the units used in table 1 and throughout this section. The complete study will be presented elsewhere.

The example we will further consider here is related to only a small part of the data obtained for the mentioned study (since it is enough for us to illustrate the correction method). We will use the MetLuc activity values corresponding to five vectors containing incrementally deleted parts of the *Mtch1* promoter (denoted by pMAN12, pMAN17, pMAN18, pMAN19 and pMAN20) as well as a control vector containing the *MetLuc* gene but no promoter region at all (pMetLuc–). The measured MetLuc activity values (the quantity 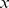 in this example) for the six vectors (or the systems) in three assays are presented in table 1 in the Introduction. This is our starting point.

### The Problem with the Results

As we advanced in the Introduction, the problem with the data in table 1 began to emerge when we computed the average of 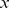 for the system 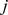, namely summed the results of all the assays divided by the total number of assays 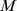:
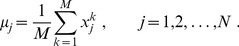
(1)


The corresponding standard deviation is computed as usual through:
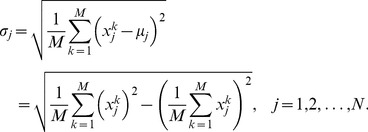
(2)


These two values were represented for all systems in the last two columns of table 1, and we could see there that the standard deviations were so large that they rendered the results almost useless. The same problem could be appreciated by looking at fig. 1 (in the Introduction) at the bar chart associated to the last two columns of table 1.

In a more quantitative way and advancing the requirement that the inter-system variation of 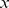 is what really matters to us, we calculated the probability that the observed difference between two average values, 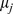 and 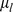 (corresponding to two different vectors) could be produced by pure chance, i.e., without the need to resort to any supplementary explanation such as the difference in the sequences of the two promoter regions in the vectors. This probability can be obtained as the so-called 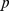-value associated to a two-sample Student’s 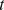-test with unequal variances, see, e.g., p. 181 in [8], and p. 253 in [9]. One usually considers the observed difference to be statistically significant when 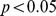, that is, when the probability that it can be obtained by pure chance is less than 5% [10]. In table 2, we present the 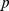-values associated to the activity measures of each pair of vectors in table 1, as computed by Microsoft Excel. We can appreciate that our intuition about the poor quality of our results is confirmed: Only two out of the fifteen possible pairs came close to the 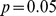 threshold, none is below it, and several are significantly larger.

**Table 2:** p-values associated to the starting results.

	pMAN12	pMAN17	pMAN18	pMAN19	pMAN20	pMetLuc–
pMAN12	–	0.475	0.198	0.662	0.349	0.161
pMAN17	–	–	0.178	0.399	0.618	0.121
pMAN18	–	–	–	0.246	0.077	0.145
pMAN19	–	–	–	–	0.340	0.215
pMAN20	–	–	–	–	–	0.050
pMetLuc–	–	–	–	–	–	–

Probabilities (or 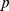-values) that the observed differences between the averages 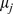 and 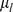 of the measured promoter activity (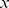 quantity) for each pair of systems (vectors) can be produced by pure chance. Values smaller than 0.05 indicate that the observed difference is statistically significant.

It is at this point when we are tempted to think that everything is lost and just throw in the towel. Our results are bad. We have to dump them and perform the experiments again. Period.

However, as we advanced in the Introduction, there are *two characteristics* about the problem we are considering here that, when combined, can save our day.

### Requirements to Apply the Correction Method

The first one is related to the type of questions we are interested in making and answering:

We are not interested in the *absolute value* of 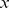 for each given system (the MetLuc activity for each vector). What really matters to us is the *variation* in 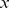 from one system to another.

For example, whether or not we could safely claim that the activity corresponding to pMAN12 is larger than, and approximately the double of that associated to pMAN20. Indeed, if we *are* interested in the absolute value of MetLuc activity in RLU, the results in table 1 are just beyond rescue and the discussion ends here.

The second characteristic together with the one we have just discussed will allow us to correct the bad looking results in table 1 and has to do with the properties of the observed measures themselves:

Even if large differences in absolute value are observed across the different assays, the ‘tendency’ of the variations is similarly captured in all three of them. Technically, the different assays present *high linear correlation* with one another.

This is even more apparent in the graphical representation in fig. 2 (in the Introduction), and without this kind of behavior in our data the correction method we will introduce next would not yield satisfactory results.

**Figure 3:**
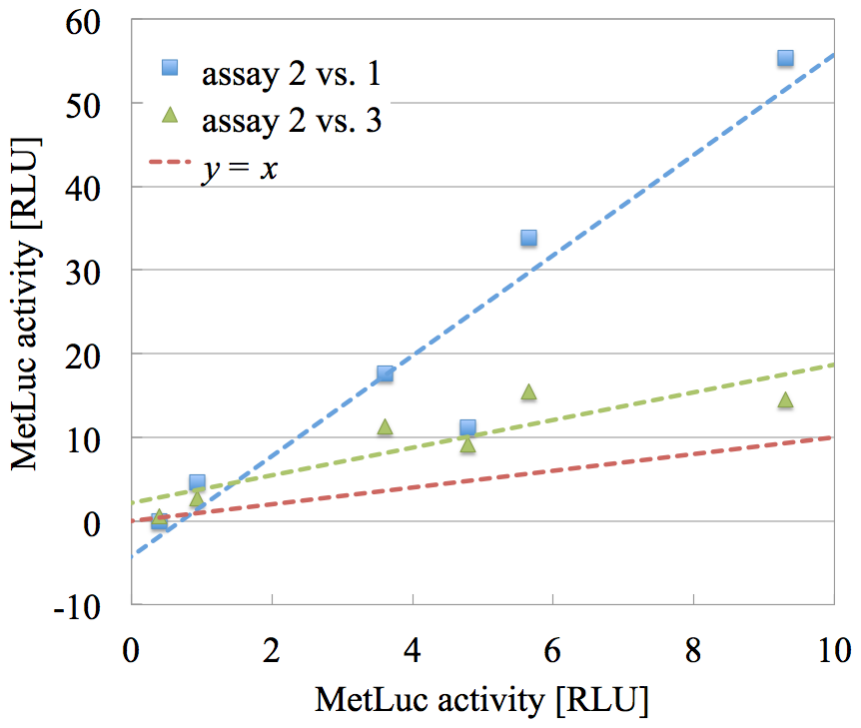
Linear correlation between assays in the starting results. Scatter plots comparing the 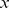 quantity (MetLuc activity) of the six systems (vectors) in table 1 for different pairs of assays. Using blue squares, MetLuc activity in assay 2 vs. the same quantity in assay 1. Using green triangles, assay 2 vs. assay 3. The least-squares fit lines are depicted using the same color as the respective points, and we also show the 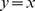 line in red for reference.

In fig. 3, we have represented two scatter plots: both using the values of assay 2 in the 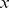-axis, one of them using the values of assay 1 as the 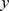-coordinate (blue squares), the other using the values of assay 3 (green triangles). We have performed the two corresponding linear fits and we have depicted the corresponding tendency lines using the same color as the respective points. We also show the 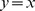 line in red for reference. For the reason behind the choice of these two concrete pairs of assays, see The method.

Several points are worth making about this graph:

- As we guessed, the linear correlation between the values in the different pairs of assays is high, with Pearson’s correlation coefficient 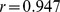 for assay 2 vs. assay 1, 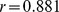 for assays 2 vs. assay 3. This is the mathematical property that embodies the intuitive property that “different assays similarly capture the tendency in the measured data”. And as we mentioned before, this high correlation is one of the two requirements for our method to be applicable.
- The fact that the fit lines present a *non-unit slope* is telling us that, although the tendency is similar across assays, the absolute value is not. The two things combined mean that among the pairs of assays there is a multiplicative systematic error and can be removed.
- The fact that the fit lines have a *non-zero intercept* is telling us that we also have an *additive multiplicative systematic error* that can be eliminated by applying our method.

### The Method

To quantitatively assess the possibility that the data in table 1 (or the corresponding one in any other experiment having the structure as described in Experimental setup) satisfies the second requirement in the previous section) and can therefore be corrected, we begin by performing all least-squares linear fits between all possible pairs of assays 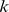 and 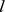 [see, e.g., p. 70 in [11]]. For each pair we use the values of the first assay for the 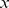 coordinate and those of the second one for the 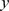 coordinate. The result of such a fit is a *tendency line* of the form:
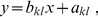
(3)where 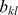 is called the *slope* and 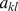 the *intercept* (or 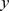-*intercept*). They are computed using the following formulas:



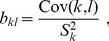
(4a)

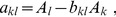
(4b)where 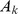 is the average of the measured quantity across systems and in the one single assay 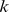 [not to be confused with the averages across assays for one single system computed using eq. (1), and presented in table 1 and fig. 1]:
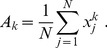
(5)Of course, 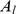 is obtained just changing 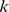 by 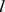 in this expression.

The quantity 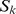 is the standard deviation in 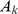, given by [compare now with eq. (2)]:
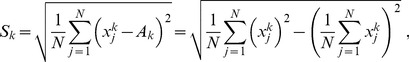
(6)and 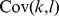 is the *covariance* between the values in assay 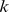 and those in assay 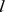:



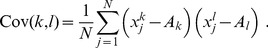
(7)With these quantities in hand, we are prepared to compute the *Pearson correlation coefficient* 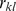 associated to the goodness of the linear fit between each pair of assays 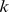 and 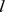, which is given by eq. (5.62) in [11]:
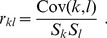
(8)


In the first three columns of table 3, we present the Pearson correlation coefficients corresponding to each pair of assays in the example experiment whose results can be read in table 1. We can see that 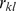 is close to 1.0 for all pairs, and we can therefore suspect that our correction method will produce sizable improvements in the data.

**Table 3:** Pearson’s correlation coefficient between the assays in the starting results.

	assay 1	assay 2	assay 3	*r_k_*
assay 1	0.000	0.947	0.852	0.900
assay 2	–	0.00	0.881	0.914
assay 3	–	–	0.000	0.867

Pearson’s correlation coefficient 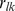 between each pair of assays in the experiment described in The experiment. The last column displays the average 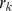 of each assay with respect to all the rest of them.

The first step to actually apply the method consists of selecting a *reference assay*. Since we do not know the ‘true’ values of the 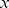 quantity (MetLuc activity) for the different systems, we will compare all the assays to the reference one and we will correct them against it.

In order to perform the selection of the reference assay with the least bias possible, we measure ‘how different’ each assay is to the rest and we choose the one that is the least different; in a sense, the most representative one. To quantify this ‘difference’ we use in fact the Pearson correlation coefficient, since it presents a property which makes it very convenient for our purposes: It discounts (is insensitive to) the possible existence of both additive and multiplicative systematic errors between the compared assays, thus measuring the difference in the variation tendency only [12]; which is exactly what we need. Also notice that, as a simple consequence of its definition in eq. (8), 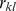 is symmetric under the permutation of the indices 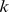 and 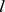. This is intuitive, since it means that ‘the difference between assays 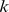 and 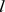
’ is the same as ‘the difference between assays 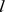 and 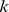
’.

The step that remains to be able to select the reference assay is simple: Just compute the *average correlation coefficient* 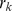 of the 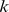-th assay with respect to all the rest of them:
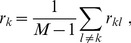
(9)and pick the one with the *largest* 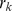.

In the last column of table 3, we show the average correlation coefficient 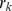 associated to each assay. We can see that it is the largest for assay 2. Therefore, we select assay 2 as our reference assay in the example we are discussing (which, by the way, explains the particular fits portrayed in fig. 3).

Now that the reference assay has been chosen and all the linear fits have been computed, we are ready to apply the *correction* to the rest of assays. If we denote by 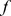 the value of the index 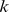 that corresponds to the reference assay (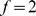 in our example) and we use 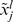 for the corrected value associated to the original quantity 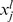 (system 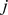, assay 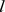), the *correction formula* reads like this:
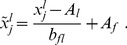
(10)


In order to produce the whole set of corrected results, we should apply this for all assays 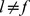, with 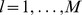, and for all systems with the index 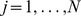.

In order to understand the reason behind this formula, it is convenient to write the inverse transformation by solving for 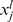:
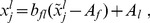
(11)and also to notice that the systems-average of 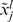 is given by:
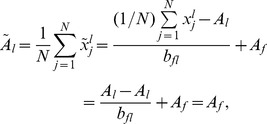
(12)i.e., all the averages of the corrected assays are equal to the average of the reference one. Now, if we take eq. (11) to the covariance in eq. (7) with 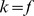, we obtain:
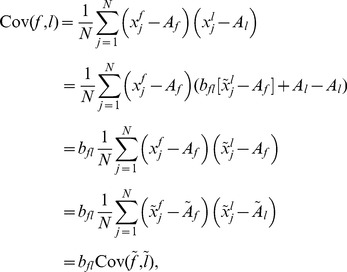
(13)


where, in the last step of the second line, we have used that 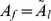 [as we proved in eq. (12)], but also that the correction in eq. (10) is obviously the identity for the reference assay 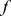 (it suffices to notice that 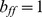), which makes 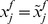, as well as all the derived quantities, such as 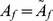. In the last line of eq. (13), we have simply used the natural notation 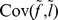 to indicate the covariance between the corrected assays 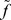 and 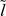. Finally, if we use eq. (13) together with the definition of the slope in eq. (4a) (with 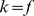), we obtain:
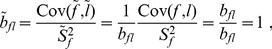
(14)where we have denoted by 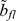 the slope associated to the fit between the corrected assays 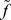 and 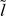, and we have used that 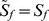. Also, it is easy to prove that:



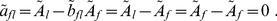
(15)That is, the slope of the fits among the corrected assays is 1 and the intercept is 0. Since we argued that the first can be interpreted as a multiplicative systematic error and the second as an additive one, we have just proved that our proposed correction in eq. (10) has the promised effect of eliminating both errors. To see that this also has the effect of reducing the standard deviations and improving the statistical significance of our results, we turn to the next section.

But before, let us mention a final consistency property of the correction method: In mathematical jargon, it is *idempotent*. In plain words, applying it twice is the same as applying it once, i.e., if we apply the whole correction process to the corrected results, we find that nothing changes. The corrected-corrected results are just the corrected results.

All the formulae needed to compute the linear fits, the inter-assay correlation coefficients, as well as the correction in eq. (10) are provided in this section and they are very simple. The reader can choose to implement them in any spreadsheet of her liking, or she can use the Perl scripts we have written for the occasion and which can be found in file S1. Also in file S2, we provide a cheat sheet with the bare steps of our method, conveniently organized, briefly stated, and stripped off of all the explanatory text that surrounds the steps in this article.

## Results

If we apply the correction in eq. (10) to our original results in table 1, we obtain the corrected values in the second part of tab. 4 (where we have repeated the uncorrected data to facilitate the comparison).

**Table 4:** Starting and corrected results.

Before						
	assay 1	assay 2	assay 3	*μ*	±	*σ*
pMAN12	33.88	5.65	15.53	18.36	±	14.33
pMAN17	17.60	3.61	11.29	10.83		7.01
pMAN18	4.62	0.94	2.72	2.76		1.84
pMAN19	55.35	9.30	14.52	26.39	±	25.22
pMAN20	11.15	4.78	9.10	8.35	±	3.52
pMetLuc–	0.00	0.39	0.54	0.31	±	0.28
**After**						
	**assay 1**	**assay 2**	**assay 3**	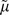	±	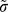
pMAN12	6.35	5.65	8.10	6.70	±	1.26
pMAN17	3.64	3.61	5.52	4.26	±	1.10
pMAN18	1.48	0.94	0.34	0.92	±	0.57
pMAN19	9.93	9.30	7.48	8.91	±	1.27
pMAN20	2.56	4.78	4.20	3.85	±	1.15
pMetLuc–	0.71	0.39	−0.98	0.04	±	0.90

Activity of the MetLuc protein under the control of six different promoter sequences measured in three assays, before and after the correction described in The method. The last two columns correspond to the average 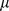 of the three assays for each vector, and the associated standard deviation (or error) 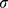. The units as well as the rest of the experiment’s details are described in the text.

**Figure 4:**
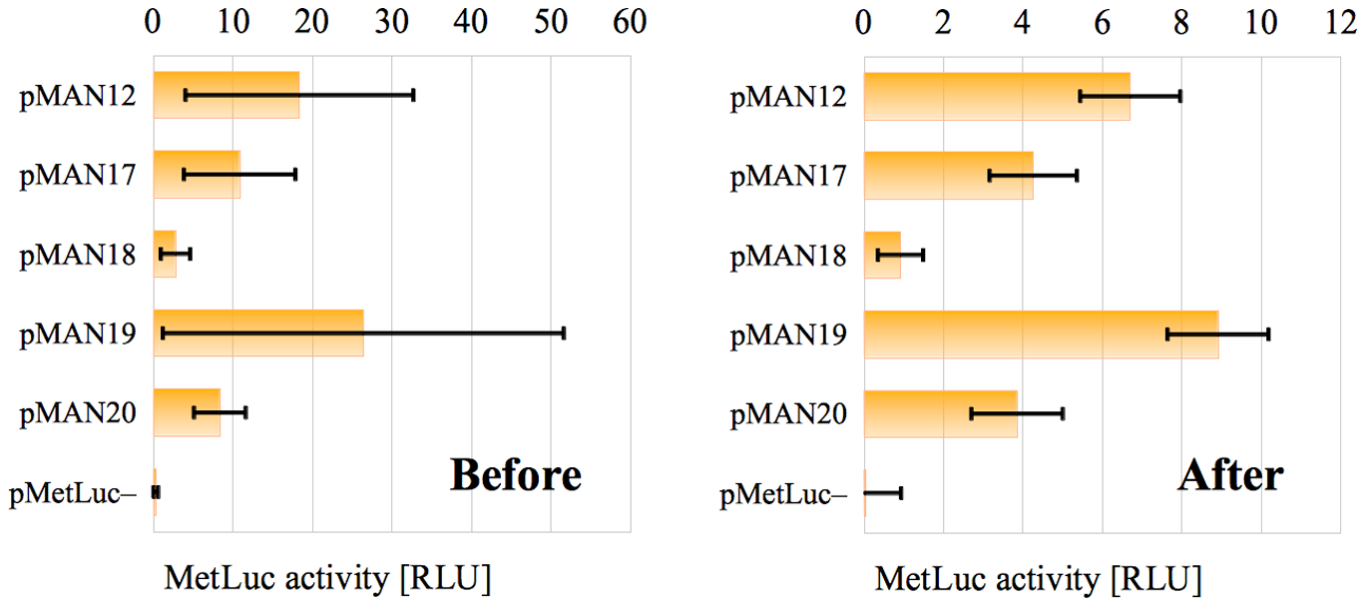
Errors in the starting and corrected results. Bar chart representation of the average values 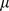 (orange bars) and the associated standard deviation 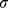 (black capped lines) in table 4, before and after the correction described in The method.

At first sight, the corrected standard deviations 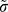 seem much better when compared to their associated averages 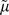 for each system. This impression is reinforced if we take a look at the corresponding bar charts in fig. 4.

If we want to be rather quantitative, and recall that the inter-system variation of MetLuc activity is what really matters to us, we can repeat the 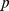-values calculation in The problem with the results for the corrected data. In tab. 5, we present both the original 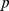-values obtained from the uncorrected results as well as the new ones. We remind the reader that the 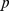-value’s meaning is that it quantifies the probability that the observed difference between two average values, 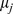 and 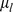, corresponding to two different vectors can be produced by pure chance, i.e., without the need to resort to any supplementary explanation such as the difference in the sequences of the two promoter regions in the vectors. One typically considers the observed difference to be statistically significant when 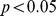, that is, when the probability that it can be obtained by pure chance is less than 5%. As we can see in tab. 5, while the original situation was despairing, with two out of the fifteen possible pairs close to the 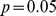 threshold, none below it, and several significantly larger, the corrected 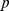-values show a much better behavior. For the corrected data, eleven out of the fifteen possible comparisons are below the 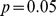 threshold, two of them are close to it, and only two are significantly larger. This means that most of the observed differences in MetLuc activity are now statistically significant.

**Table 5:** p-values associated to the starting and corrected results.

Before						
	pMAN12	pMAN17	pMAN18	pMAN19	pMAN20	pMetLuc–
pMAN12	–	0.475	0.198	0.662	0.349	0.161
pMAN17	–	–	0.178	0.399	0.618	0.121
pMAN18	–	–	–	0.246	0.077	0.145
pMAN19	–	–	–	–	0.340	0.215
pMAN20	–	–	–	–	–	0.050
pMetLuc–	–	–	–	–	–	–
**After**						
	**pMAN12**	**pMAN17**	**pMAN18**	**pMAN19**	**pMAN20**	**pMetLuc–**
pMAN12	–	0.066	**0.007**	0.100	**0.045**	**0.003**
pMAN17	–	–	**0.018**	**0.009**	0.679	**0.007**
pMAN18	–	–	–	**0.003**	**0.030**	0.236
pMAN19	–	–	–	–	**0.007**	**0.001**
pMAN20	–	–	–	–	–	**0.012**
pMetLuc–	–	–	–	–	–	–

Probabilities (or 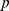-values) that the observed differences between the averages 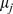 and 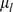 of the measured promoter activity for each pair of vectors can be produced by pure chance. The two tables correspond to the data before and after the correction described in The method. Values smaller than 0.05 indicate that the observed difference is statistically significant in both cases, and the entries satisfying this condition have been highlighted using boldface fonts.

**Figure 5:**
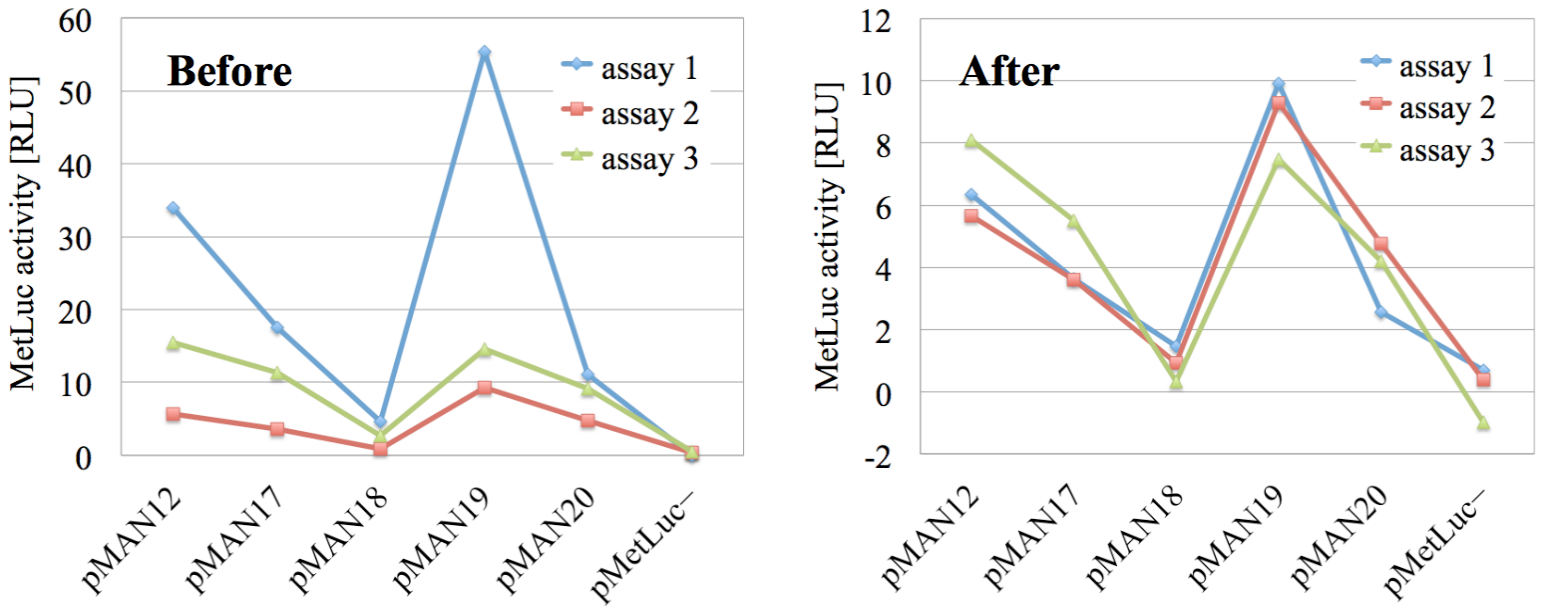
Tendency in the starting and corrected results. Variation of the quantity 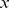 (MetLuc activity) in table 1 for the six systems (vectors) studied, before and after the correction described in The method. Each color corresponds to a different assay, and the lines joining the experimental points have been added for visual comfort.

In order to enrich our picture of what is going on here, we can also take a look at the corrected version of the tendency plot that we presented before in fig. 2 and which we now repeat here on the left of fig. 5. As we can see in the corrected tendency plot on the right, the fact that all three assays correctly captured the overall variation tendency of the data has been maximally leveraged by the correction in eq. (10). Without altering the legitimate random noise in the original results, the additive and multiplicative systematic errors have been eliminated, and the corrected tendency lines are now optimally superimposed.

**Figure 6:**
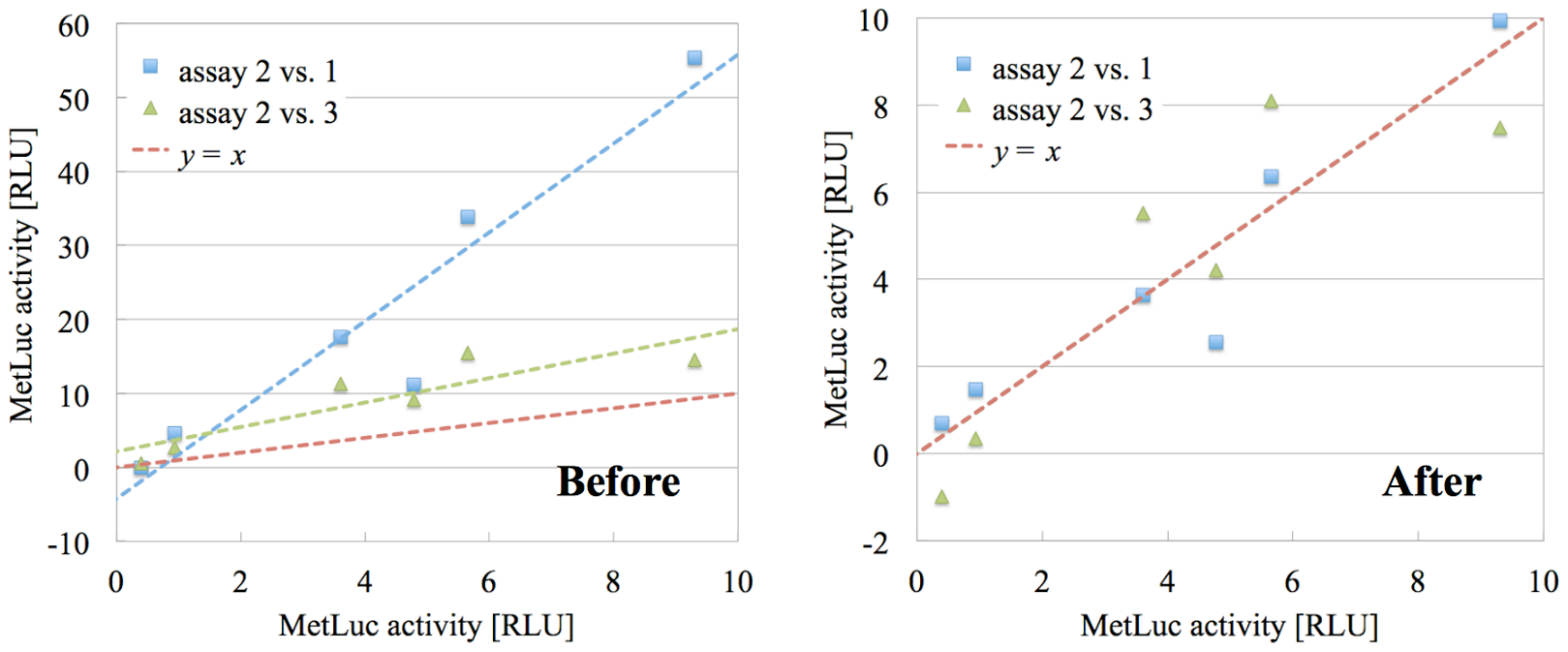
Linear correlation between assays in the starting and corrected results. Scatter plots comparing the 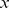 quantity (MetLuc activity) of the six systems (vectors) in table 4 for different pairs of assays, before and after the correction described in The method. Using blue squares: MetLuc activity in assay 2 vs. the same quantity in assay 1. Using green triangles: assay 2 vs. assay 3. The least-squares fit lines are depicted using the same color as the respective points, and we also show the 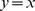 line in red for reference.

Similarly, we can compare the original and corrected scatter plots in fig. 6. In the second one, the best fit lines corresponding to assays 2 vs. 1 and assays 2 vs. 3 have been omitted because they coincide with the zero-intercept unit-slope 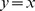 line. This is the precise mathematical embodiment of the fact that the correction in eq. (10) eliminates the additive and multiplicative errors’: it transforms all the fits against the reference assay from non-zero intercept and non-unit slope to zero intercept and unit slope. The fact that the random error is unmodified can be appreciated by the remaining dispersion of the scatter plot points with respect to the 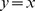 line in the second graph in fig. 6.

## Discussion

We have introduced a simple method for correcting results in multi-assay experiments under two very basic conditions, namely, only inter-system variations matter to us, and the different assays present high linear correlation with one another. The method allows to considerably reduce the standard deviation of the systems’ averages across assays, consequently increasing the statistical significance of the results. We have applied the correction method to a real experiment in cell biology where great improvements have been appreciated.

Our interpretation of the situation is that uncontrolled differences (errors) appear when a given experiment is repeated. Some of them are random (i.e., we see no pattern in them) and therefore cannot be eliminated. Some others are systematic and can be. If we represent a scatter plot where the results of one assay are placed on the 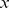-axis, the results of a different one are placed on the 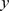-axis, and we perform a linear fit, we can expect to observe two different situations:

- The best fit line has zero 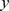-intercept and a unit slope. We interpret this as all the error being random, and no correcting action can be applied here. The data must be used ‘as is’.
- The best fit line has a non-zero intercept, a non-unit slope or both. We interpret this as some of the error being systematic, some of it random. The non-zero intercept signals an additive systematic error, the non-unit slope a multiplicative systematic one, and the dispersion of the scatter plot points from the fit line signals the part of the error that is random. In such a case, we can apply the correction in eq. (10), thus eliminating both systematic components and reducing the situation to the one described in the previous point.

As it is always the case with systematic errors, one might or might not know the actual reasons behind them (we left the apparatus on too much time, the cell number was larger than usual, we inadvertently used the wrong pipette, etc.), but we do not really need to know the reasons to confidently assert that a systematic error is indeed there. If the difference between two assays is (mostly) captured by multiplying the results of one of them by a number 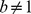 and adding a number 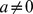, we are entitled to entertain the strong suspicion that some very real causes are behind this predictable pattern. Hence, even if we do not know these causes, it would be a wasted opportunity not to apply the correction in eq. (10). If you do know the causes, good for you. So much the better. In fact, by applying the reasoning associated to the method described here, the presence of a non-zero intercept or a non-unit slope in the fits of the different pairs of assays (plus a high linear correlation among them) may suggest to the experimenter that some additive or multiplicative systematic error is being made from assay to assay. With this clue, she can then proceed to look for the actual experimental causes behind them (in case they were previously unknown).

Also notice that systematic errors might not end at the linear order. The relation between the results of two different assays could be well described by a quadratic relation 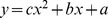 plus some random error for example; or even by higher order polynomials. No *a priori* reason can reject this possibility, however, a treatment of these more complicated cases is outside the scope of this work.

An important part of the method introduced here is that, since we do not know the ‘real’ absolute value of the measured quantities (and in fact it does not matter to us), we have to choose a *reference assay* to fit all the rest of assays to. The most reasonable way to perform this choice in an unbiased manner is to select the most representative assay in the experiment, the one that is ‘most similar to all the others’. We make this condition precise by measuring the difference of every assay to all the rest of them and choosing the one that is the least different to all the others. We use the Pearson correlation coefficient associated to the goodness of the linear fit for this purpose because it correctly discounts the additive and multiplicative systematic errors.

It is also worth mentioning that the use of the word ‘error’ for differences among one specific assay and the rest of them might seem unorthodox at first sight. After all, the ‘error’ is ideally defined as the difference between the measured quantities and their ‘real’ values. However, we think that this apparent overuse of the term is just that: apparent. Since the ‘real’ values are never actually known, the ideal definition of ‘error’ is philosophically appealing but practically inapplicable. What researchers *always* do is to compare one set of measures to some more accurate ones (but not ‘real’ yet), to some theoretical prediction (not ‘real’ either), etc. In this sense, and given that the ‘real’ values of the quantity 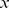 are unknown in our experimental setup described in Methods (as in all setups!), the ‘best’ guess we a priori have (before the proposed correction) of the most accurate set of measures is precisely the most representative of our assays, i.e., the one that is the least different from the rest. This is why we choose it as the reference to which all the rest of the assays are compared, and this is why the observed differences deserve to be intuitively called ‘errors’.

Although our facts come straightforward, we have found in the literature only one related proposal for a correction method that could be compared to ours (even if its rationale is never clearly expressed as we do here). This related method readily comes to mind and it consists of dividing, in each assay, the value of 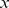 for all systems by the value of one of them. For example, we could select pMAN18 as our *normalizing vector*, divide the activities of all the vectors in each assay by the activity of pMAN18 in the same assay, and thus obtain a new set of results now expressed as a *normalized fold change in activity* with respect to the pMAN18 value (which now becomes 1.0). This is used for example in [13]–[16] or fig. S3 in [17].

The result of applying this normalization to the original data in tab. 6 is presented in tab. 6. We see that the standard deviations have been reduced and in fact the overall improvement is similar to what we obtained when applying the correction method introduced in this work. However, this normalization procedure presents some drawbacks which, in our opinion, render it inferior to our method. Namely:

- It demands an arbitrary choice (that of the normalizing system) which seems ad hoc and prevents automatization in some degree. And related to this, it does not seem easy to interpret nor does it seem completely legitimate that the corrected result for the normalizing system has a zero standard deviation.
- If we recall the general formula for the propagation of errors in p. 50 of [11],
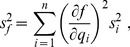
(16)where 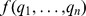 is a function of 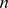 random variables with standard deviations (errors) 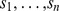, we can use it to compute the error in the normalized quantity 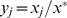, where 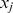 is the measured result for the system 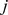 (in a given assay) and 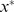 is the quantity measured for the system chosen to normalize the results:

**Table 6:** Results normalized via division by the values of one system.

	assay 1	assay 2	assay 3	*μ*	±	*σ*
pMAN12	7.33	6.01	5.71	6.35	±	0.86
pMAN17	3.81	3.84	4.15	3.93	±	0.19
pMAN18	1.00	1.00	1.00	1.00	±	0.00
pMAN19	11.98	9.89	5.34	9.07	±	3.40
pMAN20	2.41	5.09	3.35	3.62	±	1.36
pMetLuc–	0.00	0.41	0.20	0.20	±	0.21

Fold change in activity of the MetLuc protein under the control of six different promoter sequences measured in three assays. The numbers in this table have been obtained from the activity data in table 1 through division by the value for pMAN18.



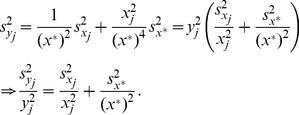
(17)We see that the error in the normalized quantity 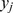 relative to the value of 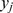 itself is the sum of the relative errors of 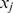 and 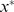. Now, if we happen to choose a particular normalizing system with high relative error, this could spoil the whole assay when we divide all the results by 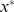, even if the rest of measures were accurate.

- The described normalizing procedure seems fit to eliminate multiplicative systematic errors, but not additive ones.

Our method suffers from none of these problems:

- No choice of a ‘special’ normalizing system is needed. (There is a choice of a reference assay, but it is made in a justified way, as we have explained.)
- In a manner of speaking, it distributes the normalization among all the values in a given assay, thus minimizing the probability that one specially bad apple spoils the whole basket.
- It eliminates both multiplicative and additive systematic errors.

If we check out exhaustive textbooks in biostatistics, such as [8], [9], [18], or more wide ranging ones, such as [19]–[23], we do not find any account of a correcting method that is similar to that we propose here. Sometimes the texts come close but never hit the target.

One way of coming close is discussing *repeated measures*. See for example chap. 9 in [21], chap. 27 in [20], or chap. 8 in [24], [18], and p. 346 [8] for detailed discussions of such concept in biosciences.

A very similar experimental setup to the one used here (and thoroughly described in Experimental setup (i.e., measuring the same quantity on 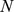 systems and repeating the experiment 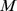 times) is the “Repeated measures” one, but with a fundamental difference attached to it, as it tackles measurements *that are expected to change from repetition to repetition* [e.g., a time series, or table II of [1] discussed in Experimental setup]. In turn, in our setup, results of several repetitions are expected to be the same, and this is key to our further decision to correct them, a rather unnatural take in the repeated-measures setup. A second essential difference adds up to it, and that is, for the “repeated measures” setup, the interest lies not only with the inter-system variation but with the absolute value as well, while in ours, the absolute value does not matter.

Another similar situation to the one we have considered here is dealt with in In p. 539 of [19], namely *blocking*. However, they do not discuss what to do if there is an obvious linear correlation between the blocks (as in their figure 13.6a) or take any correction action on it (as shown from their example in figure 13.12).

One of the reasons we can offer for not finding any previous references in the literature to a method as straightforward as ours (for as much as we have inquired) could be the usual interpretation of the range of application of the least-squares fit protocol. That is typically fitting some values in the 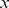-axis against those on the 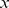-axis and uses them as such to assess a possible linear relationship between *two different quantities*, let’s say apples and oranges. So much so that 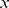 is typically called the *independent variable*, while 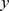 is the dependent one. It is a key conceptual step in our approach to realize that it actually makes sense to investigate the linear correlation of some quantity *against itself* (as measured in two different assays), and consequently interpret any difference between the two as an experimental error (in the manner we explained above).

Another reason that could possibly be behind the absence of any precedents is the fact that despite being quite intuitive to us, systematic errors of the multiplicative kind are very rarely discussed in such literature as they are normally considered to be additive.

After a thorough search we have only found anecdotal mentions in a paper that discusses the influence of natural fires on the air pollution of the Moscow area [25], in a proceedings paper about anticorrosion coating [26], in a recent work concerned with calibration of spectrographs for detecting earth-mass planets around sun-like stars [27], and in a similar paper focused in the detection and study of quasars [28]. All authors consider the possibility of a multiplicative systematic error in their models or measurements but take no action to correct it.

Discussions on multiplicative systematic errors are given more room in papers by [29] (in p. 3), who acknowledge the existence of multiplicative systematic errors in the context of analytical chemistry and the necessity to eliminate them or by [30] who discusses the possibility of both additive and multiplicative systematic errors, as well as their respective relation with non-zero 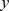-intercepts and non-unit slopes. Finally, in p. 39 of [11], the authors discuss multiplicative systematic errors (which they call *gain shifts* or *gain errors*) and also provide several examples where this multiplicative systematic error can appear, but none of the above-mentioned authors provide any method for eliminating them.

It is also worth mentioning that, in [30] and in [11], the authors consider the error to be defined with respect to “true” results (to calibrate experimental protocols) or at least more accurate results (to calibrate measuring devices). As we have explained above (when discussed the choice of the reference assay), our perspective on the issue is different, and so it is the approach. That is why, if we want to correct our results against some “better” data, we are presumably interested not only in the variations of the measured quantity, but also in its absolute value.

We have only found one work, concerned with gas electron diffraction data [31], in which the authors *both* consider the existence of multiplicative systematic errors *and* take actions to correct them. However, the proposed correction is particular to the concrete problem studied, and the experimental setup is different to the one described in Experimental setup: The authors refer to systematic errors in experimental data with respect to the “true” values, not to systematic errors between different measures of the same quantity as we do here.

It is also worth mentioning that sometimes scientists choose to show a “representative” assay in which the trend among the different systems is apparent but the Pearson correlation is not strong between pairs of assays. This could be justified in a qualitative way and based on the knowledge of the experiment by the scientist. However, application of our method to such a situation would not be legitimate since one of the fundamental conditions needed for that is not satisfied.

## Conclusions

We have introduced a method for correcting the data in experiments in which a single quantity 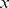 is measured for a number of systems in multiple repetitions or assays. If we are not interested in the absolute value of 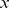 but in the inter-system variations only, and if the results in different assays are highly correlated with one another, we can use the proposed method to eliminate both additive and systematic differences (errors) between each one of the assays and a suitably chosen reference one. As we have shown, using a real example of a cell biology experiment, such correction can considerably reduce the standard deviation in the systems’ averages across assays, and consequently improve the statistical significance of the data.

The method is of a very wide applicability to experimental results and very likely to numerical simulations as well, (as long as the structure of the setup and the requirements on the data are those mentioned and carefully discussed in Experimental setup). This, together with its simplicity of application (the only mathematical infrastructure needed to apply it is basically least-squares linear fits), makes the method of very wide interest in any quantitative scientific field that deals with data subject to uncertainty.

Some possible lines of future work include the application of the method to a wider variety of problems, a deeper statistical analysis of its properties and the assumptions behind it, as well as the extension to systematic differences of higher-than-linear order (that we briefly mentioned in Discussion).

## Supporting Information

File S1 **Perl scripts for correcting the experimental data as described in this article.** Compressed zip file including the scripts, example data files, and a README file explaining basic installation and usage.
(ZIP)

File S2 **Cheat sheet.** Summary of the experimental setup, the possible problem with the data, the requirements to apply the correcting method, and the method itself. Quick reference for the reader.
(PDF)
