# Supplementary material for: Reducing the Standard Deviation in Multiple-Assay Experiments Where the Variation Matters but the Absolute Value Does Not
Source: PLoS One. 2013 Oct 30;8(10):e78205. doi: 10.1371/journal.pone.0078205 (PMC3813515; doi:10.1371/journal.pone.0078205)
Supplement: File S2 — Cheat sheet. Summary of the experimental setup, the possible problem with the data, the requirements to apply the correcting method, and the method itself. Quick reference for the reader. (PDF) [file pone.0078205.s002.pdf]

Reducing the standard deviation in multiple-assay experiments where the variation matters but the absolute value does not

Cheat sheet

Supplementary material of the paper at: http://arxiv.org/abs/1309.2462

September 11, 2013

Experimental setup

You have N systems, among which a specific one may be called *system* j, with $j = 1, 2, \ldots, N$. You measure a quantity x for each one of the N systems, and you repeat M times the whole set of N measures. A generic repetition is termed *assay* k, with $k = 1, 2, \ldots, M$. An example for $N = 6$, $M = 3$ is shown in tab. 1. It is also convenient to use x_j^k to denote the value of the quantity x measured for system j in the k-th assay (e.g., in tab. 1, $x_4^2 = 4.78$).

	assay 1	assay 2	assay 3	$\mu \pm \sigma$
system 1	33.88	5.65	15.53	18.36 ± 14.33
system 2	17.60	3.61	11.29	10.83 ± 7.01
system 3	4.62	0.94	2.72	2.76 ± 1.84
system 4	55.35	9.30	14.52	26.39 ± 25.22
system 5	11.15	4.78	9.10	8.35 ± 3.52
system 6	0.00	0.39	0.54	0.31 ± 0.28

Table 1: 6×3 values of a quantity x, measured for six different systems in three assays. The last two columns correspond to the average μ of the three assays for each system, and the associated standard deviation (or error) σ. The units are irrelevant for the discussion.

The different systems can be anything, from cities to DNA sequences, from people to chunks of metal. They can even be the same system at different times if the quantity x is expected to evolve in some reproducible manner. The differences among the assays could be due to the experiments being performed by the same researcher on different days, by different (but in principle equally skilled) researchers using the same equipment, by the same researcher using different (but in principle equally accurate) equipment, by different (but in principle equally proficient) laboratories, etc. As long as we expect different assays to yield the same results.

The problem

The standard deviation from the systems' averages across assays in tab. 1 is comparable to the average itself for most of the systems. Only on a couple of them you are 'lucky' enough so that the former is about half the value of the latter. You check the corresponding chart in fig. 1, and you see the same despairing situation. The error bars are humongous, and this will render your results statistically insignificant if you perform, for example, a Student's t-test to check whether or not the observed differences are real.

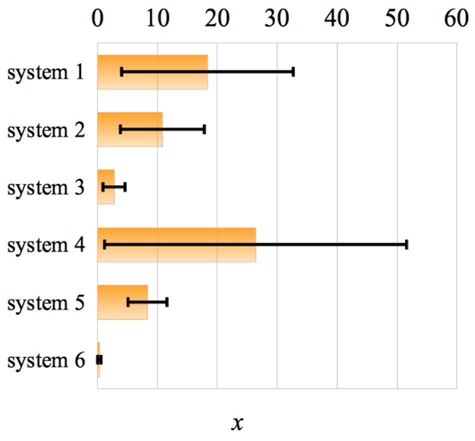

Figure 1: Bar chart representation of the average values μ (orange bars) and the associated standard deviation σ (black capped lines) in tab. 1. The units are irrelevant for the discussion.

The requirements

If two requirements about your problem and your results are met, you can apply the correction method in the next section to reduce the standard deviations and increase the statistical significance of your data:

- The *absolute value* of x for each given system is not really very important to you. What you are really interested in properly measuring is the *variation* in x from one system to another. For example, whether or not you could safely claim that the value of x corresponding to system 1 is larger than, and approximately the double of, that associated to system 5.

- Even if you seem to be measuring huge differences in absolute value across the different assays, the 'tendency' of the variations is similarly captured in all of them. You can check this by looking at a graphical representation of your data such as the one in fig. 2, or you could be safer and check for high linear correlation between each pair

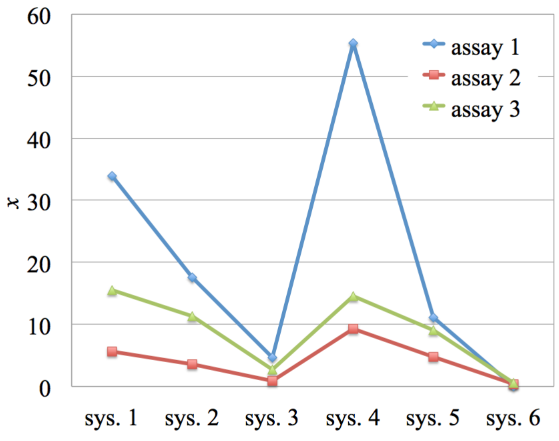

Figure 2: Variation of the quantity x in tab. 1 for the six systems studied. Each color corresponds to a different assay, and the lines joining the experimental points have been added for visual comfort.

of assays by performing a number of least-square linear fits (placing one array in the x-axis and the other one in the pair in the y-axis).

The correction method

Perform a linear fit for every pair of assays k and l, with $k \neq l$, and $k = 1, \ldots, N$, placing assay k in the x-axis and assay l in the y-axis.

For this, compute the averages, A_k and A_l, of the measured quantity across systems for each assay in the pair:

$$A_k = \frac{1}{N} \sum_{j=1}^{N} x_j^k \ . \tag{1}$$

Of course, A_l is obtained just changing k by l in this expression.

Compute the standard deviation in A_k (and A_l):

$$S_k = \sqrt{\frac{1}{N} \sum_{j=1}^{N} \left(x_j^k - A_k\right)^2} = \sqrt{\frac{1}{N} \sum_{j=1}^{N} \left(x_j^k\right)^2 - \left(\frac{1}{N} \sum_{j=1}^{N} x_j^k\right)^2} \ . \tag{2}$$

Compute the covariance between the values in assay k and those in assay l:

$$\mathrm{Cov}(k,l) = \frac{1}{N} \sum_{j=1}^{N} \left(x_j^k - A_k\right) \left(x_j^l - A_l\right) \ . \tag{3}$$

Take these quantities to the slope b_{kl} and the intercept a_{kl}:

$$b_{kl} = \frac{\mathrm{Cov}(k,l)}{S_k^2} \ , \tag{4a}$$

$$a_{kl} = A_l - b_{kl} A_k \ , \tag{4b}$$

defining the best fit line:

$$y = b_{kl} x + a_{kl} \ . \tag{5}$$

The results of these fits allow you to check for the required high linear correlation mentioned in the previous section. This is done by computing the Pearson's correlation coefficient for every pair of assays k and l:

$$r_{kl} = \frac{\mathrm{Cov}(k,l)}{S_k S_l} \ . \tag{6}$$

In the first three columns of tab. 2, we can see that r_{kl} is close to 1.0 for all pairs in tab. 1. We can therefore suspect that our correction method will produce sizable improvements in the data.

Now, for each k compute the average correlation coefficient r_k of the k-th assay with respect to all the rest of them:

$$r_k = \frac{1}{M-1} \sum_{l \neq k} r_{kl} \ , \tag{7}$$

and pick the one with the *largest* r_k as the *reference assay*, i.e., the one against all the other assays will be corrected. The values for the example in tab. 1 are presented in the last column of tab. 2. We see that, in this case, the reference assay is the second one.

	assay 1	assay 2	assay 3	r_k
assay 1	0.000	0.947	0.852	0.900
assay 2	—	0.00	0.881	0.914
assay 3	—	—	0.000	0.867

Table 2: Pearson's correlation coefficient r_{lk} between each pair of assays in tab. 1. The last column displays the average r_k of each assay with respect to all the rest of them.

Finally, denote by f the value of the index k that corresponds to the reference assay ($f = 2$ in our example) and use $\tilde{x}_j^l$ for the corrected value associated to the original quantity x_j^l (system j, assay l). Now, the *correction formula* reads like this:

$$\tilde{x}_j^l = \frac{x_j^l - A_l}{b_{fl}} + A_f \ . \tag{8}$$

In order to produce the whole set of corrected results, you should apply this for all assays $l \neq f$, with $l = 1, \ldots, M$, and for all systems with the index $j = 1, \ldots, N$.

In tab. 3 and fig. 3, we show the numerical values and the bar charts for the corrected results obtained from the example in tab. 1 through the application of the correction in eq. (8).

	assay 1	assay 2	assay 3	$\tilde{\mu} \pm \tilde{\sigma}$
system 1	6.35	5.65	8.10	6.70 ± 1.26
system 2	3.64	3.61	5.52	4.26 ± 1.10
system 3	1.48	0.94	0.34	0.92 ± 0.57
system 4	9.93	9.30	7.48	8.91 ± 1.27
system 5	2.56	4.78	4.20	3.85 ± 1.15
system 6	0.71	0.39	−0.98	0.04 ± 0.90

Table 3: Corrected values of the quantity x resulting from applying the correction method to the original measures in tab. 1.

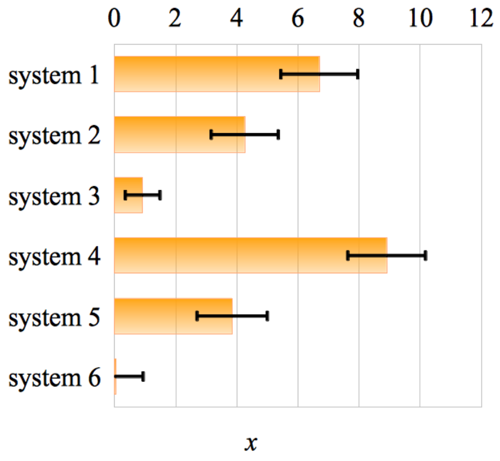

Figure 3: Bar chart representation of the corrected average values $\tilde{\mu}$ (orange bars) and the associated standard deviation $\tilde{\sigma}$ (black capped lines) in tab. 3.

As you can see the standard deviations as well as the associated statistical significance have greatly improved. If your data fits into the basic setup and satisfies the requirements, you will probably see a similar improvement. Enjoy!

All the formulae needed to compute the linear fits, the inter-assay correlation coefficients, as well as the correction in eq. (8) are provided in this section and they are very simple. The reader can choose to implement them in any spreadsheet of her liking, or she can use the Perl scripts we have written for the occasion and which can be found here.

For more information, check the complete article at: http://arxiv.org/abs/1309.2462
